# Using host genetics to infer the global spread and evolutionary history of HCV subtype 3a

**DOI:** 10.1093/ve/veab065

**Published:** 2021-07-09

**Authors:** Shang-Kuan Lin, Nicola De Maio, Vincent Pedergnana, Chieh-Hsi Wu, Julien Thézé, Daniel J Wilson, Eleanor Barnes, M Azim Ansari

**Affiliations:** Nuffield Department of Population Health, Big Data Institute, University of Oxford, Li Ka Shing Centre for Health Information and Discovery, Old Road Campus, Oxford OX3 7LF, UK; European Molecular Biology Laboratory, European Bioinformatics Institute (EMBL-EBI), Wellcome Genome Campus, Hinxton CB10 1SD, UK; MIVEGEC, Université de Montpellier, CNRS, 911 avenue Agropolis, Montpellier 34000, France; Building 54, Mathematical Sciences University of Southampton, Highfield, Southampton SO17 1BJ, UK; Department of Zoology, University of Oxford, South Parks Road, Oxford, Oxfordshire OX1 3PS, UK; Université Clermont Auvergne, INRAE, VetAgro Sup, UMR EPIA, Centre INRAE Clermont-Auvergne-Rhône-Alpes, Saint-Genès-Champanelle 63122, France; Nuffield Department of Population Health, Big Data Institute, University of Oxford, Li Ka Shing Centre for Health Information and Discovery, Old Road Campus, Oxford OX3 7LF, UK; Peter Medawar Building for Pathogen Research, University of Oxford, South Parks Road, Oxford, Oxfordshire OX1 3SY, UK; Nuffield Department of Population Health, Big Data Institute, University of Oxford, Li Ka Shing Centre for Health Information and Discovery, Old Road Campus, Oxford OX3 7LF, UK; Peter Medawar Building for Pathogen Research, University of Oxford, South Parks Road, Oxford, Oxfordshire OX1 3SY, UK

**Keywords:** HCV, evolution, phylogeography, phylogenetics, host–virus genetics

## Abstract

Studies have shown that hepatitis C virus subtype 3a (HCV-3a) is likely to have been circulating in South Asia before its global spread. However, the time and route of this dissemination remain unclear. For the first time, we generated host and virus genome-wide data for more than 500 patients infected with HCV-3a from the UK, North America, Australia, and New Zealand. We used the host genomic data to infer the ancestry of the patients and used this information to investigate the epidemic history of HCV-3a. We observed that viruses from hosts of South Asian ancestry clustered together near the root of the tree, irrespective of the sampling country, and that they were more diverse than viruses from other host ancestries. We hypothesized that South Asian hosts are more likely to have been infected in South Asia and used the inferred host ancestries to distinguish between the location where the infection was acquired and where the sample was taken. Next, we inferred that three independent transmission events resulted in the spread of the virus from South Asia to the UK, North America, and Oceania. This initial spread happened during or soon after the end of World War II. This was subsequently followed by many independent transmissions between the UK, North America, and Oceania. Using both host and virus genomic information can be highly informative in studying the virus epidemic history, especially in the context of chronic infections where migration histories need to be accounted for.

## 1. Introduction

It is estimated that 71 million people are infected with the hepatitis C virus (HCV) worldwide, many in the developing world (WHO 2017). Following the acute phase of infection, the majority of the infected individuals enter a chronic asymptomatic phase of infection that can last for decades. Globally, chronic HCV infection is one of the leading causes of liver cirrhosis and hepatocellular carcinoma (HCC) (El-Serag 2012). While there are currently no vaccines for HCV, the recently developed direct-acting antivirals have significantly improved the safety and efficacy of treatment regimens for HCV infection (Smith et al., 2021).

HCV is highly diverse and is currently classified into eight major genotypes (denoted by numbers 1–8), each of which has been divided into many subtypes (denoted by lower case letters, e.g. 1a, 1b, etc.). The clinical outcomes of chronic HCV infection are influenced by viral genetics. For instance, it has been shown that HCV genotype 3 is associated with a higher risk of developing HCC (Nkontchou et al., 2011; Kanwal et al., 2014; El-Serag et al., 2016; Lee et al., 2019) and with higher rates of direct-acting antiviral treatment failures (Jacobson et al., 2013; Lawitz et al., 2013). Furthermore, amino acid variation in the NS5A protein is associated with viral load (Ansari et al., 2017, 2019), and amino acid variation in the core protein is associated with the development of HCC (Sedeño-Monge et al., 2017).

The various genotypes and subtypes of HCV are associated with distinct epidemiological and geographical patterns of distribution. Some HCV genotypesid="aq7 have limited geographical distribution, such as genotypes 4,id="aq7 5, and 6, which are found mainly in North and Central Africa,id="aq7 Southern Africa, and East and Southeast Asia, respectivelyid="aq7 (Messina et al., 2015). HCV genotypes 1 and 3 (specifically subtypesid="aq7 1a, 1b, and 3a) are globally distributed and are the mostid="aq7 common cause of HCV infections worldwide (Messina et al., 2015). id="aq7Phylogenetic studies have inferred the potential origin for someid="aq7 of these globally distributed subtypes. HCV genotype 1 isolatesid="aq7 sampled from Central and West Africa have a much higher genetic diversity than those sampled from the other parts of the world (Jeannel et al., 1998; Ndjomou, Pybus, and Matz 2003), indicating a long-term endemicity in the region, followed by the global spread of subtypes 1a and 1b. For HCV genotype 3, studies have found the virus to be highly diverse in South and Southeast Asia, indicating the origin of subtype 3a (Tokita et al., 1994; Mellor et al., 1995). Various studies have also inferred the evolutionary and epidemiological history of the globally spread subtypes (Verbeeck et al., 2006; Njouom et al., 2007; Magiorkinis et al., 2009; Zehender et al., 2013; Choudhary et al., 2014; McNaughton et al., 2015; Paraskevis et al., 2019). Most of these studies have used a limited number of samples and have focused on specific genomic regions (such as Core, E1/E2, and NS5A/NS5B) and/or on the epidemiological history of a restricted geographic region.

Generating virus whole genomes and host genome-wide genotyping data for 507 patients located in the UK, Canada, USA, Australia, and New Zealand from the BOSON is the name of the clinical trial. This name is not a shortened version of any specific phrase. Gilead which sponsored this trial was using names like “FISSION”, “FUSION” and “POSITRON” for their sofosbuvir trials BOSON cohort (Foster et al., 2015), we investigated the epidemiological history and global spread of HCV subtype 3a (HCV-3a). Using the host genome-wide genotyping data to infer the ancestry of the patients, we observed that almost all of the viruses from the patients with South Asian ancestry clustered near the root of the phylogenetic tree irrespective of the country the patient came from. A simple explanation for this observation is that patients of South Asian ancestry are more likely to have been infected in South Asia rather than their country of residence. We combined host ancestry and virus sequence data to infer whether a South Asian ancestry patient was infected in South Asia or his/her country of residence by performing a structured coalescent analysis applied to the inferred ancestries. The results were then used to perform a phylogeographic analysis of HCV-3a that distinguished between these two groups. Accounting for this confounder, we observed that three independent transmission events resulted in the spread of the virus from South Asia to the UK, North America, and Oceania. We also inferred that these initial spreads happened during or soon after the end of World War II.

For the first time, we have used host genetic information to inform virus phylogeographic analysis and to distinguish between the location of the host at the time of sampling and the location where the infection was acquired, which may be different in the context of chronic infections.

## 2. Results

### 2.1 Molecular clock signal of HCV-3a sequences

Our 507 HCV-3a whole genomes from the BOSON study lack a molecular clock signal (Supplementary Fig. S1) as they were collected during a short interval in the 2013–14 period (Foster et al., 2015). To resolve this, we compiled 42 previously published HCV-3a whole genomes from public databases, the earliest of which was sampled in Canada in 1991. To explore the temporal signal in the updated data set, we estimated a maximum likelihood (ML) tree from whole genomes and calculated the correlation between root-to-tip genetic distance and sampling dates of the sequences using TempEst (Supplementary Fig. S1) (Rambaut et al., 2016). We estimated a substitution rate of 2.13 × 10^−3^ (95 per cent CI: 1.61 × 10^−3^ to 2.64 × 10^−3^) per site per year, which is consistent with the previous estimate for HCV-3a (1.65 × 10^−3^ substitutions per site per year, with 95 per cent HPD: 1.19 × 10^−3^ to 2.14 × 10^−3^; McNaughton et al., 2015). We also employed the Bayesian evaluation of temporal signal (BETS) analysis on our updated data set (Duchene et al., 2020) to formally test for the strength of the temporal signal. BETS estimates a Bayes factor comparing a model with the actual sampling times to a model in which the samples are constrained to be contemporaneous. The model with the actual sampling times was a better fit to our data with a log Bayes factor of 26 in favour of it.

### 2.2 Enrichment of South Asian ancestry individuals among HCV-3a-infected individuals from the UK

**Figure 1.**
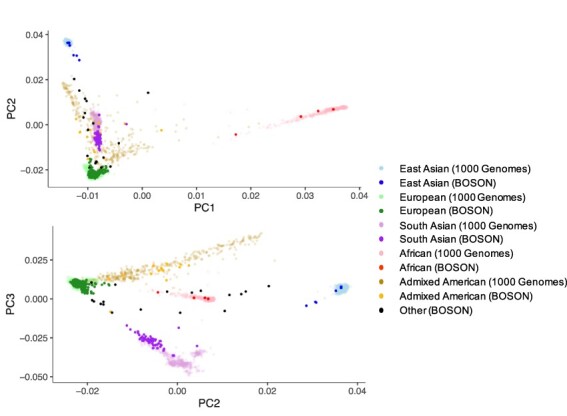
Projection of the host genetic PCs of BOSON cohort onto the 1000 Genome Project PCs to detect and validate self-reported ancestries. Each dot is an individual, and the colours indicate host ancestries. The 1000 Genome Project data were used to adjust the self-reported ethnicities in the BOSON cohort.

To determine the host ancestry of the patients in our study, we estimated principal components (PCs) of the host genome-wide genotyping data for the BOSON cohort and projected them onto the genetic PCs of the 1000 Genomes Project where the ancestries are known (The 1000 Genomes Project Consortium 2015). We used these projections to validate and adjust the self-reported host ancestry of the patients (Fig. 1, Supplementary Fig. S2, see Section 4) and to distinguish between South Asian and East Asian ancestry, which both were self-reported as Asian in our data set. For BOSON hosts with HCV-3a infection where genetic data were not available, host ancestries were designated as their self-reported ancestries (nineteen individuals). The host ancestries of HCV-3a sequences downloaded from public databases (where no host genetic data were available) were assigned based on the majority ethnic group of the countries where the samples came from. We observed that the majority of the patients had white European ancestry (*N* = 433, 80 per cent), and South Asians were the second largest group (*N* = 69, 13 per cent). The other host ancestries present in this data set were Admixed Americans (*N* = 12), East Asians (*N* = 6), Africans (*N* = 4), and other ethnic groups (*N* = 16).

Although the patients in the BOSON cohort were recruited from the UK (*N* = 211), the USA (*N* = 65), Canada (*N* = 64), Australia (*N* = 113), and New Zealand (*N* = 35) where the majority of the population are of white European ancestry, we observed an enrichment of patients of South Asian ancestry in this cohort, especially in the UK where South Asian ancestry patients made 19 per cent of the HCV-3a infections (41/211, *P*-value = 1.4 × 10^−13^), while making up only 5 per cent of the population (Office for National Statistics; National Records of Scotland; Northern Ireland Statistics and Research Agency, 2016).

### 2.3 Distinct epidemiological history of HCV-3a among South Asians living in the West

**Figure 2.**
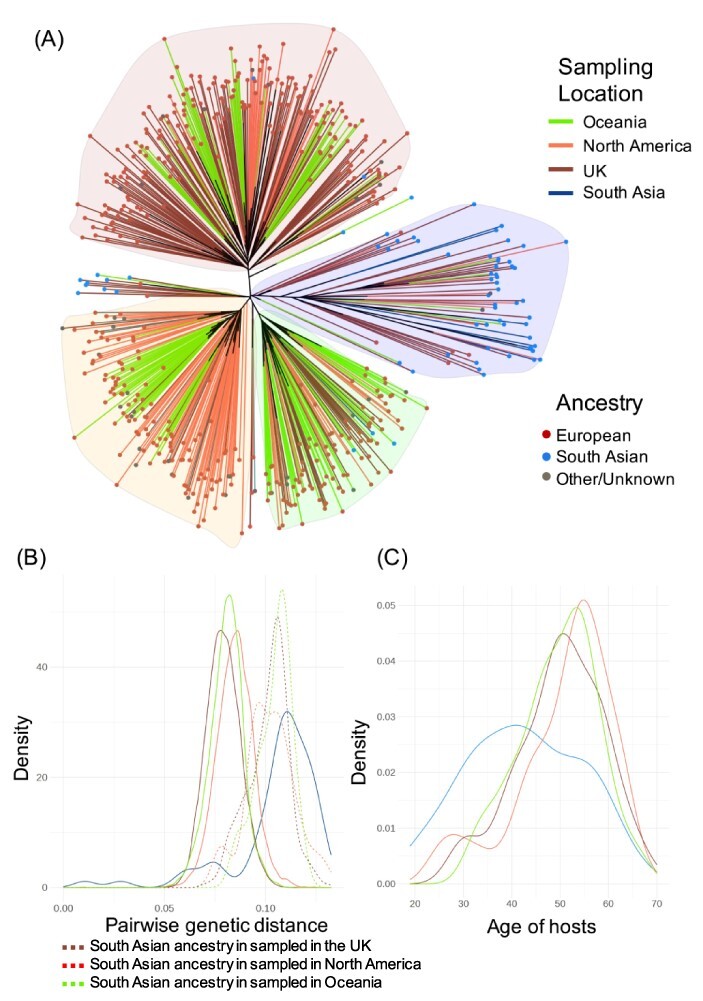
HCV-3a phylogeny and its association with host ancestry and demography. (A) ML tree with the terminal branches coloured according to the sampling location and the tip marker indicating the host ancestry as inferred from host genetic data. (B) Distribution of pairwise genetic distances between HCV whole genomes in different host groups. (C) Age distribution in different host groups (age data only available for hosts from the BOSON Cohort).

To investigate the co-variation between host ancestries and virus sequence data, we used RAxML (Stamatakis 2014) to estimate an ML tree from the viral whole genomes and looked for associations between the virus tree and host ancestries (Fig. 2A). The tree consists of four major clades. Surprisingly, we discovered that almost all of the viruses from individuals of South Asian ancestry in the BOSON cohort, irrespective of which country they came from (UK, USA, Canada, Australia, or New Zealand), grouped in one clade with South Asian sequences downloaded from public repositories.

**Figure 3.**
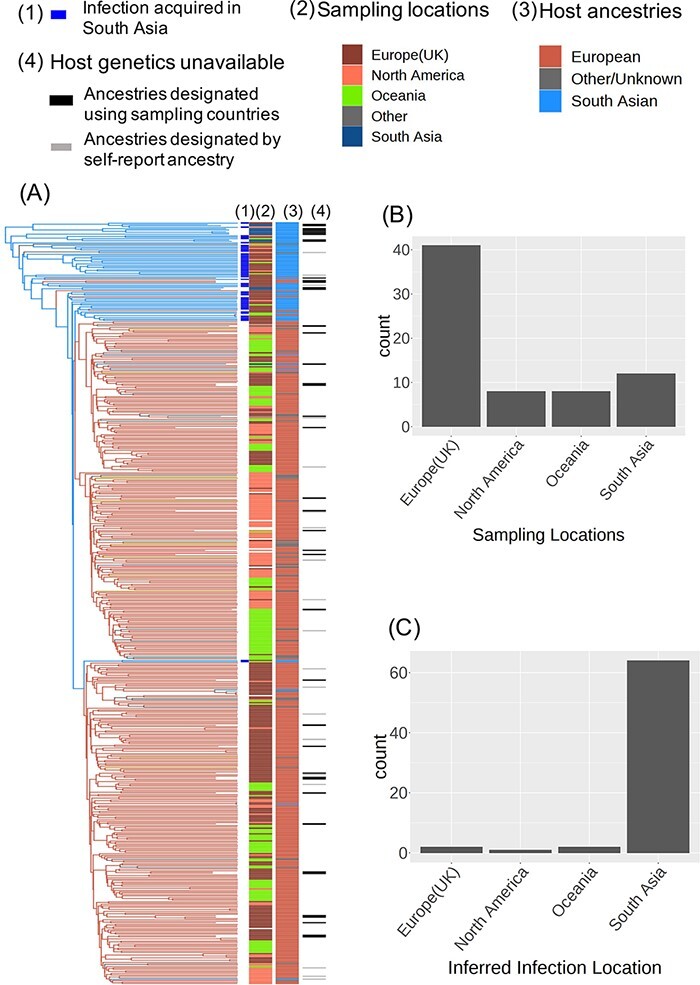
Detection of the location of infection for South Asian hosts. (A) A structured coalescent phylogeographic analysis applied to a time-calibrated ML tree and the host ancestries to infer the location of infection for South Asian hosts. The branches are coloured by the most likely host ancestral state. Lines on the tips of the trees indicate (1) South Asian hosts sampled in a Western country and inferred to have been infected in South Asia, (2) sampling locations, (3) host ancestries, and (4) absence of host genetic information. The two bar charts represent the location distribution for (B) sampling locations and (C) inferred locations of infection among South Asian hosts living in the West.

To explain this observation, we hypothesized that individuals of South Asian ancestry are more likely to have been infected in South Asia either due to travel history or acquiring the infection before migration to the West. Under this hypothesis and assuming that South Asia is the origin of the epidemic, we expect to observe a higher viral genetic diversity among South Asian hosts regardless of the country they come from and for these isolates to coalesce near the root of the tree and nest the isolates from individuals with other ancestries. Using pairwise sequence distances to measure genetic diversity, we observed that the mean viral pairwise distance for South Asian hosts in each of the UK, North America, and Oceania region is higher than hosts with other ethnicities in these regions and similar to viral genetic diversity observed in South Asia (Fig. 2B). Additionally, South Asian hosts in western countries are on average younger than hosts with other ethnicities in these countries (Fig. 2C), indicating a distinct epidemiological history. We also inferred a time-calibrated ML tree and observed that the majority of HCVs from individuals with South Asian ancestry living in the Western countries coalesce near the root of the tree and nest the isolates from individuals with other ancestries (Fig. 3A).

### 2.4 Inferring where the infection was acquired for South Asians living in the West

Using sampling location for South Asian hosts in a phylogeographic analysis will bias the analysis as the infection is likely to have been acquired in South Asia rather than the country of residence. To detect the location of infection for South Asian hosts living in the West, we applied structured coalescent analysis, as implemented in MASCOT (Müller, Rasmussen, and Stadler 2018), to host ancestries (see Section 4). Any HCV-3a isolate from a South Asian host in a Western country where all its ancestral nodes were also estimated to have South Asian ancestry were inferred to have infected its host in South Asia instead of the country of residence (Fig. 3A). The locations of infection for other isolates were set to be the same as their sampling locations. This resulted in 52 South Asian hosts sampled in Western countries inferred as having been infected in South Asia and five inferred as having been infected in their sampling countries (Fig. 3B, C). For the rest of the paper, we will use these inferred locations of infection rather than the country of sampling for the South Asian hosts living in the West.

### 2.5 Phylogeographic analysis of HCV-3a whole genomes to trace its global spread

**Figure 4.**
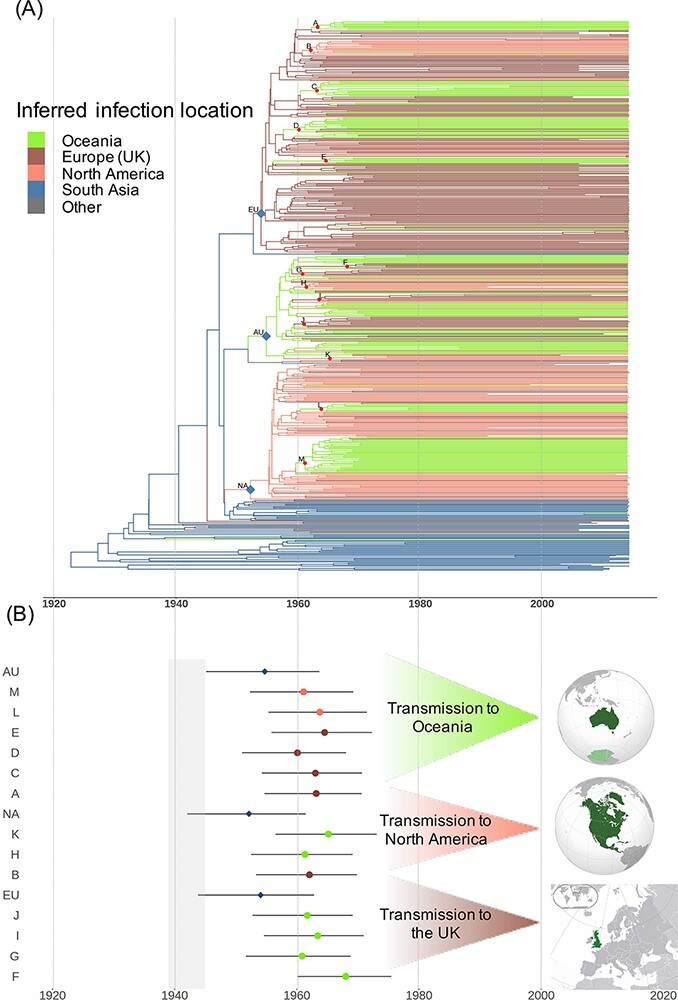
Phylogeographic analysis of the global spread of HCV-3a. (A) Maximum Clade Credibility (MCC) tree with branches colour-coded by the most likely infection location. The three nodes that correspond to the earliest introductions from South Asia to the UK (EU), North America (NA), and Oceania (AU) are indicated using blue diamonds. Other significant transmission events between continents are indicated using red circles. (B) The 95 per cent HPD of the time of the highlighted nodes in (A). The estimated time points are colour-coded by the geographical source of transmission. The grey block corresponds to the time period of World War II (1939–45).

We used Bayesian Evolutionary Analysis Sampling Trees (BEAST) to infer a maximum clade credibility time-calibrated phylogeny of the virus whole genomes using a relaxed molecular clock model (Drummond et al., 2006; Suchard et al., 2018). The resulting estimated substitution rate was 1.69 × 10^−3^ (95 per cent Highest posterior density (HPD): 1.41 × 10^−3^ −1.96 × 10^−3^) substitutions per site per year. Apart from the South Asian isolates near the root, the tree consists of three major clades, the largest of which contains mainly sequences from the UK (63 per cent UK samples, node EU on Fig. 4A, posterior probability >99 per cent). The next biggest clade contains sequences primarily from North America (55 per cent North American samples, node NA, posterior probability 99 per cent). The largest fraction of the samples in the third clade come from Oceania (42 per cent Australia and New Zealand samples, node AU, posterior probability >99 per cent). Within each major clade, we observed the geographical structuring of the isolates. For instance, subclades containing predominantly Oceanian isolates are dispersed across the three clades, indicating a complex phylogeographical history with multiple independent introductions (Fig. 4A). The same pattern is true for North American isolates and to a lesser degree for the UK isolates.

We conducted a structured coalescent phylogeographic analysis using the maximum clade credibility tree generated by BEAST and applied it to the inferred locations of infection for South Asians living in the West and the location of sampling for individuals of other ancestries. We inferred the time to the most recent common ancestor (TMRCA) of the HCV-3a isolates in our study to be 1923 (95 per cent HPD, 1905–38), and the geographic origin is estimated to be in South Asia (geographic posterior probability >0.99) where HCV-3a is likely to have been endemic. Subsequently, three distinct lineages escaped South Asia and spread globally at around the same time. These independent transmissions were from South Asia to the UK around 1954 (95 per cent HPD: 1946–63, node EU in Fig. 4A), to North America about 1952 (95 per cent HPD:1942–61, node NA in Fig. 4A), and to Oceania around 1955 (95 per cent HPD: 1945–63, node AU in Fig. 4A), and the posterior probability of ancestral of state reconstruction for all three nodes was 1 (Supplementary Table S2). These three introductions form the three major clades dominating different continents (UK, North America, and Oceania). All these introductions are estimated to have occurred during or shortly after World War II. Following these early transmissions out of South Asia, there were frequent independent transmissions between continents until the late 1960s. Large numbers of independent transmission events to and from Oceania (Australia and New Zealand) are observed during this period (Fig. 4B, Supplementary Table S2).

## 3. Discussion

We report the first study that incorporates host genetic information to investigate the genetic epidemiology of a virus (HCV-3a). This additional layer of information allowed us to infer host ancestries and to discover that viruses from South Asian ancestry hosts cluster together and coalesce near the root of the phylogenetic tree regardless of the country they were collected in (all the samples in the BOSON cohort came from the UK, North America, and Oceania and not South Asia). We also observed more virus genetic diversity among South Asian ancestry hosts in these regions than other ancestries. Hosts with South Asian ancestry also had a different age distribution from hosts with other ancestries and were over-represented among HCV-3a-infected individuals in the UK and other regions.

A possible explanation is that these hosts were infected within the South Asian community in their country of residence. However, this scenario is not supported by the data since under this hypothesis we would expect the viral genetic diversity within each of these South Asian communities (in the UK, North America, and Oceania) to be significantly lower than that sampled from South Asia and to potentially observe distinct South Asian clades for each region. Both of these predictions are not supported by the data (Fig. 2A). In addition, different studies have found contradictory results regarding the significance of intrafamilial transmission as a route for HCV infection, reducing the support for the hypothesis of within-family transmission (Indolfi 2013).

The simplest hypothesis to explain these distinct patterns is that South Asian ancestry hosts in Western countries are more likely to have been infected in South Asia, which has the highest prevalence of HCV genotype 3 infection in the world (Messina 2015), either before migration or during travels to the region. This hypothesis would predict a higher viral genetic diversity among South Asian hosts regardless of the country they come from and for these isolates to coalesce near the root of the tree and nest the isolates from individuals with other ancestries, both of which are supported by the data. Additionally, studies have shown a 5-fold increased risk of HCV infection for South Asians born outside of the UK over the UK-born South Asians (Harris et al., 2012).

To infer the locations of infections for South Asian ancestry hosts living in the West, we conducted a structured coalescent analysis on host ancestries to infer their location of infection. For virus sequences obtained from public databases (where we do not have host genetic information), the host ancestry is assigned as the majority ethnic group in the sampling countries. Several of these samples are assigned white European ancestry hosts as they were collected in the West, but they cluster with the South Asian samples near the root of the tree. It is likely that these samples come from South Asian ancestry hosts but were sampled in the West. Despite this conservative approach, we observed that the coalescence events near the root of the phylogenetic tree are almost exclusively between virus isolates from South Asian ancestry hosts.

We estimated the TMRCA of HCV-3a isolates to be in 1923 (95 per cent HPD: 1905–38), which is consistent with the previous estimates for HCV-3a (Khan et al., 2009; Choudhary et al., 2014; McNaughton et al., 2015). One previous study has found the TMRCA of global HCV-3a sequences to be around 300 years ago (Zehender et al., 2013), much earlier than any other study. This study included four Pakistani isolates that formed a distinct and distant clade that increased the overall TMRCA of the whole tree. Removing these sequences, the TMRCA of the rest of the tree was 1943 (95 per cent HPD: 1885–1958), which is consistent with our and other studies. The four distant Pakistani isolates were not included in our study as they only include the NS5B region and not the whole genome.

Using phylogeographic analysis, we inferred the geographic origin of the samples in our study to be in South Asia (geographic posterior probability >0.99). The finding of a South Asian origin agrees with previous studies on the evolutionary history of HCV genotype 3 (Tokita et al., 1994; Mellor et al., 1995; Simmonds et al., 2005; Khan et al., 2009; Choudhary et al., 2014). We then inferred three independent introductions of HCV-3a from South Asia to the UK, North America (USA and Canada), and Oceania (Australia and New Zealand), which began the global spread of HCV-3a out of South Asia. All of these introductions were estimated to be in the 1950s, shortly after the end of World War II. More than 2 million South Asian soldiers participated in the war, and the virus may have been transmitted from them to soldiers from other Commonwealth countries and the Allied Forces, potentially through medical procedures such as blood transfusions and therapeutic injections in field hospitals with poor conditions (McNaughton et al., 2015). The infection would have then been transferred to the home countries after the war. Another possible route of transmission from South Asia to the UK could be the South Asian migration to the UK following World War II. British Nationality Act of 1948 enabled the migration of citizens of Commonwealth countries to Britain with very few limits, to fulfil the labour shortages that resulted from World War II (British Nationality Act 1948).

From the 1960s, a series of complex and frequent transmissions between continents followed, presumably due to the growth in global travel. In our data set, during this period, we observed at least six independent transmissions from the UK and North America to Australia, which was promoting mass immigration under the ‘populate or perish’ policy with a large number of migrants from the UK (Landon-Lane and Robertson 2002). This period also coincides with the exponential growth of the HCV genotype 3 epidemic reported in previous studies (Choudhary 2014). Various historical events have been linked during this period to the HCV expansion. This includes the rise of intravenous drug use (IDU) in Europe and North America (Stimson 1993), which has been linked to the growth of HCV subtypes 1a and 1b (Magiorkinis et al., 2009). Since IDU is shown to be the main transmission route for HCV genotype 3 (Harder et al., 2004; Pybus et al., 2005), we suspect that these events indeed facilitated the spread of the virus during this period and could have been intensified by blood transfusion and unsafe therapeutic injections (Magiorkinis et al., 2009).

In this study, we used the largest HCV-3a whole-genome data set to date and combined it with the host genetic data to reconstruct the virus epidemic history. Using the host genetic data, we discovered that almost all of the viruses from South Asian ancestry hosts cluster together near the root of the tree irrespective of which country the host came from. It also allowed us to distinguish between viruses from South Asian ancestry hosts acquired in South Asia and the country of sampling. Using these data, we observed three independent introductions from South Asia to the UK, North America, and Oceania during or soon after the end of World War II, as well as many subsequent independent transmission events between the UK, North America, and Oceania. The continuous improvement and the reduction in the cost of high throughput sequencing technologies mean that joint genomic information from host and pathogen is becoming routinely available. Such data provide an additional layer of information that can be informative in studying pathogen epidemic histories, especially chronic infections where the place the infection is acquired can be different from the current location of the host.

## 4. Methods

### 4.1 Materials

Five hundred and seven HCV-3a whole-genome sequences were obtained from the BOSON clinical trial study (Foster et al., 2015). Due to the lack of temporal signal in the BOSON data, we downloaded previously published HCV-3a sequences from the Los Alamos HCV database (Kuiken et al., 2005). The following criteria were used to filter the sequences:

1. The sequence has to be categorized as HCV-3a.
2. The sequence has to cover the coding region of the genome.
3. The sequence data have to contain a sampling date.
4. When there are multiple samples from the same patient at different time points, we only include the earliest sample.
5. When there are multiple samples from the same patient at the same time point, we randomly select one sample to include in our data set.

This resulted in forty-eight sequences that were added to our data set (Supplementary Table S1).

### 4.2 Quality control of viral sequences

The resulting 555 whole-genome sequences were aligned using MAFFT (Katoh, Rozewicki, and Yamada 2019). We then used FastTree (v2.1.10) (Price, Dehal, and Arkin 2010) to build an ML tree. TempEst (Rambaut et al., 2016) was used to explore the molecular clock signal of the data using regression to measure the correlation between the root-to-tip genetic distances and sampling times. We removed nine outliers (all of which are from the BOSON cohort) whose root-to-tip genetic distance deviated from the expectation of the linear model by more than 0.025 substitutions per site per year (Supplementary Fig. S1). The remaining 546 sequences were then realigned using MAFFT (Katoh, Rozewicki, and Yamada 2019). Hypervariable regions 1 and 2 in the sequences were removed from the aligned sequences due to their extremely high mutation rates (Simmonds et al., 2005; Lamoury et al., 2015). We then investigated the aligned sequences by looking at their ML tree as constructed by FastTree (Price, Dehal, and Arkin 2010). We observed that six pairs of sequences on the tips of the tree were very closely related (Supplementary Fig. S3). Upon further investigation, we found that for all pairs, both pairs were from the same country. Furthermore, all these samples were downloaded from Los Alamos database, had little description available in GenBank, and were submitted by the same authors (Supplementary Table S3). This raised the possibility that these pairs of closely related samples were obtained from the same individuals. To be conservative, we randomly discarded one sample in each of these closely related pairs. As a result, six more sequences were removed from our data set and the final dataset consists of 498 sequences from the BOSON cohort and 42 sequences downloaded from Los Alamos database. To formally test for the presence of molecular clock signal after the described quality control steps, we conducted a BETS analysis (Duchene et al., 2020). For this analysis, two independent Bayesian coalescent-based phylogenetic analyses were conducted using BEAST (v1.10.24) (Suchard et al., 2018). In one of the BEAST runs, the sampling dates were constrained to be contemporaneous and in the other the actual sampling dates were used. The set-up of the BEAST analyses is the same as the main phylogenetic analysis described in the later section, except that the Markov chain Monte Carlo (MCMC) was run for 10 million steps. We ran a stepping-stone sampling calculation to estimate the marginal likelihood of our models for model comparison (Xie et al., 2011).

### 4.3 Host ancestry designation

We downloaded the unimputed 1000 Genomes Project phase 3 genetic data (The 1000 Genomes Project Consortium 2015). Using Plink (v1.9) (Chang et al., 2015), the Single nucleotide polymorphisms (SNPs) in the 1000 Genomes Project were pruned by linkage disequilibrium on all autosomes, and the same set of SNPs were also pruned from the host genetic data from the BOSON cohort. PCs were then estimated for both 1000 Genomes Projects and BOSON respectively using Plink (v1.9) (Chang et al., 2015). We then projected the PCs of the BOSON cohort onto the PCs of the 1000 Genomes Project. Next, we trained a random forest classifier as implemented by R package randomForest (Liaw and Wiener 2002) on the top 20 PCs from the 1000 Genome Project. We used the trained model to predict the host ancestry for the BOSON cohort. We used a threshold of 60 per cent for the random forest score to assign ancestries in the BOSON cohort. For individuals where the random forest scores were less than 60 per cent, the ‘Other/Unknown’ ancestry was assigned. For BOSON hosts where genetic data were unavailable (*N* = 19), we used the following scheme for host ancestry designation: we inferred hosts with self-reported ethnicity of ‘Asian’ (*N* = 3) to be of South Asian ancestries as most self-reporting Asians in BOSON are of South Asian ancestries (84 per cent) according to the result of PCA; we inferred hosts self-reporting as ‘White’ to be of European ancestries (*N* = 15); and we designated hosts with other self-reported ethnicities as ‘Other/Unknown’ ancestry. For sequences that were downloaded from Los Alamos HCV database, the host genetic information was not available. The host ancestries of these samples were assigned according to the major ancestry group of the country the sample came from.

### 4.4 Phylogenetic analysis

The molecular clock and Bayesian coalescent-based phylogeny of HCV were estimated using BEAST (v1.10.4) (Suchard et al., 2018), which infers rooted and time-scaled phylogenies via an MCMC algorithm. Because of the large sample size in our data set, we took a few steps to ensure that the MCMC computation could be finished within a reasonable time. We first used FastTree (Price, Dehal, and Arkin 2010) to build an ML tree and then used TempEst (Rambaut et al., 2016) to root the tree. TempEst searches for the most likely root by finding the point in the tree that maximizes the likelihood of the tree given the sampling dates of the tips. The rooted ML tree was then used with the R package TreeDater (Volz and Frost 2017) to estimate a time-scaled tree. The resulting tree was then used as the starting point for the MCMC analysis conducted by BEAST. For the coalescent analysis, we used the HKY substitution model with a gamma rate heterogeneity model and with base frequencies set to be estimated. We also used the uncorrelated relaxed clock model with a log-normal distribution and a constant population size model for the coalescent process. The priors of the Hasegawa-Kishino-Yano (HKY) transition–transversion parameters were set to a log-normal distribution with a mean of 1 and SD of 1.25. The prior for base frequencies was set to a Dirichlet distribution with an alpha of 1. The priors for estimating the clock rate’s mean and SD were set to a continuous-time Markov chain rate reference and an exponential distribution (mean = 0.333), respectively. The prior for population size was set to be a uniform distribution between 0 and 10^100^. The ‘new tree operator mix’ option that is available in BEAUti (Drummond et al., 2012) was chosen for specifying the moves conducted by MCMC. Finally, the length of the MCMC chain was set to 100 million. MCMC analysis by BEAST was then run twice, and the result was combined. Tracer (v1.7.1) (Rambaut et al., 2018) was used to visually inspect the results of the MCMC analysis to ensure good mixing. Besides Bayesian coalescent-based phylogeny, we also used an ML approach to estimate the phylogeny of HCV in our results. RAxML (v8.2.12) (Stamatakis 2014) was used to estimate an ML tree that was then rooted with TempEst (Rambaut et al., 2016). The tree was then dated by treeDater (Volz and Frost 2017). The BEAST2 (Bouckaert et al., 2014) package MASCOT (Müller, Rasmussen, and Stadler 2018) was used for the phylogeographical analysis, where the phylogeny was fixed, and population sizes and migration rates between populations were set to be identical across all populations. Two MASCOT analyses were performed in this study. For the analysis of host ancestries, the prior on the migration rate is set to have a log-normal distribution with the underlying normal distribution having a mean of −6 and SD of 0.5. For the analysis of the infection locations, the migration rate is set to have a log-normal distribution with the underlying normal distribution having a mean of −4 and SD of 1. In both MASCOT analyses the prior on the population size is a log-normal distribution with the underlying normal distribution having a mean of 0 and SD of 2. The MCMC chain length was set to 10 million steps for both MASCOT runs.

## Supplementary Material

veab065_Supp

## Data Availability

Human genotype data are deposited in the European Genome-phenome Archive under accession code EGAS00001002324. HCV sequence data are deposited in GenBank under accession codes KY620313–KY620880. Information on access to the study data is available at http://www.stop-hcv.ox.ac.uk/data-access.
