# Supplementary material for: Using host genetics to infer the global spread and evolutionary history of HCV subtype 3a
Source: Virus Evol. 2021 Jul 9;7(2):veab065. doi: 10.1093/ve/veab065 (PMC8438900; doi:10.1093/ve/veab065)
Supplement: veab065_Supp [file veab065_supp.zip › Supplementary_File_for_Review.docx]

**Supplementary Material for the paper “Using host genetics to infer the global spread and evolutionary history of HCV subtype 3a” by Lin et. al.**


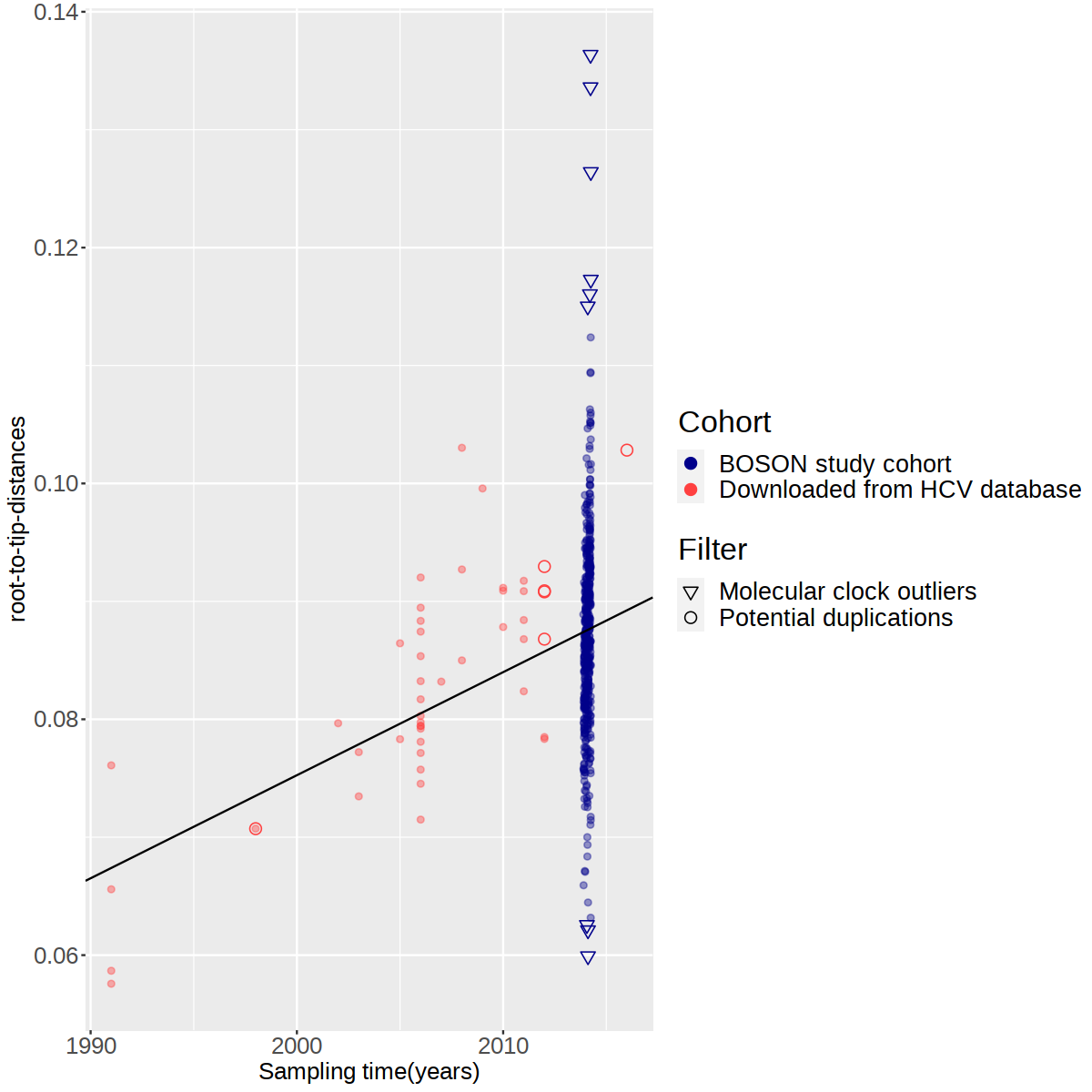


**Supplementary Figure 1**: Molecular clock analysis and the temporal distribution of HCV-3a samples. A maximum likelihood tree generated from whole genomes was used to estimate root to tip distance for each isolate which was then plotted against the sampling date and a regression line was fitted to the data using TempEst. Sequences that would be filtered in downstream quality controls are shown in different shapes.


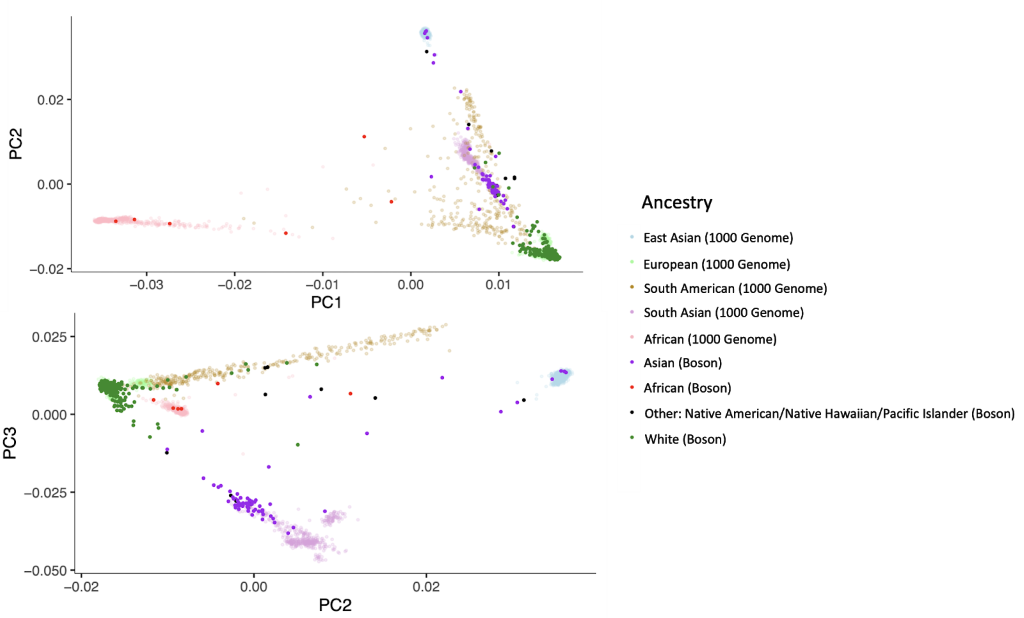


**Supplementary Figure 2**: Scatter plots of the BOSON cohort host genotypes projected onto the first three PCs calculated from the 1000 Genomes Project. The points in the plots are colored by ethnicities of individuals in the 1000 Genomes project and BOSON self-reported ethnicities.


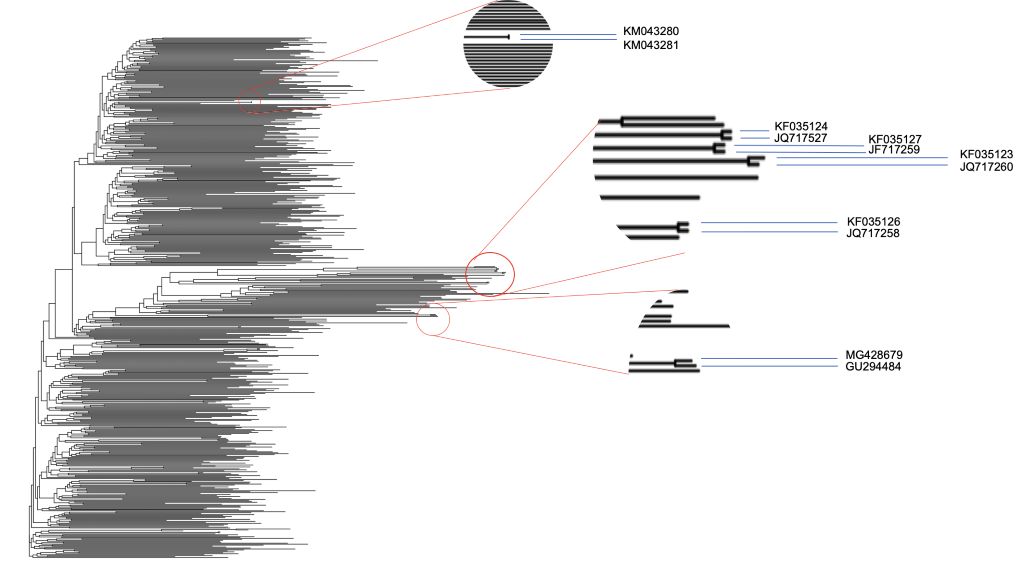


**Supplementary Figure 3**: The samples that we identified as potential duplicates, illustrated in an unrooted ML tree.

| Accession Number | QC Result | Country | Year |
| --- | --- | --- | --- |
| GQ356204 | passed | UK | 2005 |
| GQ356206 | passed | UK | 2005 |
| GQ356209 | passed | UK | 2006 |
| GQ356214 | passed | UK | 2006 |
| GQ356200 | passed | UK | 2006 |
| GQ356211 | passed | UK | 2006 |
| JF509175 | passed | UK | 2006 |
| GQ356217 | passed | UK | 2006 |
| GQ356207 | passed | UK | 2006 |
| GQ356201 | passed | UK | 2006 |
| GQ356208 | passed | UK | 2006 |
| GQ356215 | passed | UK | 2006 |
| GQ356205 | passed | UK | 2006 |
| GQ356212 | passed | UK | 2007 |
| KJ470615 | passed | Canada | 1991 |
| KJ470612 | passed | Canada | 1991 |
| KJ470613 | passed | Canada | 1991 |
| KM043280 | Potential duplications | USA | 1998 |
| KM043281 | passed | USA | 1998 |
| AY956467 | passed | USA | 2002 |
| KM587622 | passed | Australia | 2012 |
| GQ356216 | passed | UK | 2003 |
| KJ470614 | passed | Canada | 1991 |
| HQ912953 | passed | China | 2008 |
| JQ717254 | passed | India | 2010 |
| JF509177 | passed | UK | 2006 |
| GQ356203 | passed | UK | 2006 |
| KC844041 | passed | China | 2011 |
| JN588558 | passed | Pakistan | 2009 |
| GU294484 | passed | Pakistan | 2008 |
| MG428679 | Potential duplications | Pakistan | 2016 |
| GQ356213 | passed | UK | 2006 |
| GQ356202 | passed | UK | 2006 |
| JF509176 | passed | UK | 2006 |
| JQ717259 | passed | India | 2010 |
| KF035127 | Potential duplications | India | 2012 |
| JQ717257 | passed | India | 2011 |
| KF035124 | Potential duplications | India | 2012 |
| JN714194 | passed | India | 2011 |
| JQ717255 | passed | India | 2010 |
| GQ275355 | passed | India | 2003 |
| JQ717260 | passed | India | 2008 |
| KF035123 | Potential duplications | India | 2012 |
| JQ717258 | passed | India | 2011 |
| KF035126 | Potential duplications | India | 2012 |
| JQ717256 | passed | India | 2011 |
| KF035125 | passed | India | 2012 |
| GQ356210 | passed | UK | 2006 |

**Supplementary Table 1**: List of sequences included in our dataset that were downloaded from Los Almos database and quality control results.

| label | MCC posterior probability | Ancestral state posterior probability |
| --- | --- | --- |
| AU | 0.9995 | 1 |
| M | 0.9907 | 1 |
| L | 1 | 1 |
| E | 1 | 1 |
| D | 0.9995 | 1 |
| C | 0.9988 | 1 |
| A | 0.9995 | 1 |
| NA | 0.9887 | 1 |
| K | 1 | 1 |
| H | 0.9979 | 1 |
| B | 0.998 | 1 |
| EU | 0.9991 | 1 |
| J | 0.7383 | 1 |
| I | 1 | 0.99667037 |
| G | 0.9281 | 1 |
| F | 1 | 1 |

**Supplementary Table 2**: Posterior probabilities for the MCC tree and the results of MASCOT ancestral state reconstruction regarding the nodes of interest as labelled in Figure 4

| Duplicated pair | Authors | Sampling location |
| --- | --- | --- |
| MG428679 | Ahmad,S., Ali,I. and Ahmad,S. | Pakistan |
| GU294484 | Rehman,I., Butt,S., Idrees,M., Rafique,S., Akbar,H., Zubair,M., Awan,Z., Manzoor,S., H usain,A., Akram,M., Khubaib,B. and Aftab,M. | Pakistan |
| KM043280 | Stoddard,M.B., Li,H. and Shaw,G.M. | USA |
| KM043281 | Stoddard,M.B., Li,H. and Shaw,G.M. | USA |
| KF035126 | Choudhary,M.C., Natarajan,V., Mishra,G., Tripathi,R., Gupta,E. | India |
| JQ717258 | Choudhary,M.C., Mishra,G., Tripathi,R., Gupta,E., Singh,T. | India |
| JQ717260 | Choudhary,M.C., Natarajan,V., Pandey,P., Gupta,E., Sharma,S., Tripathi,R., Kumar,M. S., Kazim,S.N. and Sarin,S.K. | India |
| KF035123 | Choudhary,M.C., Natarajan,V., Mishra,G., Tripathi,R., Gupta,E., Singh,T., Trehanpati,N., Kazim,S.N., Kumar,M.S. and Sarin,S.K. | India |
| JQ717257 | Choudhary,M.C., Natarajan,V., Pandey,P., Gupta,E., Sharma,S., Tripathi,R., Kumar,M. S., Kazim,S.N. and Sarin,S.K.. | India |
| KF035124 | Choudhary,M.C., Natarajan,V., Mishra,G., Tripathi,R., Gupta,E., Singh,T., Trehanpati,N., Kazim,S.N., Kumar,M.S. and Sarin,S.K | India |
| KF035127 | Choudhary,M.C., Natarajan,V., Mishra,G., Tripathi,R., Gupta,E., Singh,T., Trehanpati,N. , Kazim,S.N., Kumar,M.S. and Sarin,S.K. | India |
| JQ717259 | Choudhary,M.C., Natarajan,V., Pandey,P., Gupta,E., Sharma,S., Tripathi,R., Kumar,M.S., Kazim,S.N. and Sarin,S.K. | India |

**Supplementary Table 3**: Detail information of potential duplicate isolates from the downloaded HCV-3a sequences.
